# G1P3 (IFI6), a mitochondrial localised antiapoptotic protein, promotes metastatic potential of breast cancer cells through mtROS

**DOI:** 10.1038/s41416-018-0137-3

**Published:** 2018-06-14

**Authors:** Venugopalan Cheriyath, Jaspreet Kaur, Anne Davenport, Ashjan Khalel, Nobel Chowdhury, Lalitha Gaddipati

**Affiliations:** 1 Department of Biological and Environmental Sciences, Texas A&M University-Commerce, Commerce, TX 75429 USA; 2 Department of Biology, Texas Woman’s University, Denton, TX 76204 USA

**Keywords:** Breast cancer, Cancer metabolism

## Abstract

**Background:**

Redox deregulations are ubiquitous in cancer cells. However, the role of mitochondrial redox deregulation in metastasis remains unclear. In breast cancer, upregulation of mitochondrial antiapoptotic protein G1P3 (IFI6) was associated with poor distance metastasis-free survival (DMFS). Therefore, we tested the hypothesis that G1P3-induced mitochondrial redox deregulation confers metastatic potentials in breast cancer cells.

**Methods:**

Cell migration and invasion assays; confocal and immunofluorescence microscopy; and Illumina HumanHT-12 BeadChip to assess gene expression.

**Results:**

Consequent to its localisation on inner-mitochondrial membrane, mtROS were higher in G1P3-expressing cells (MCF-7^G1P3^). G1P3-overexpressing cells migrated and invaded faster than the vector controls with increased number of filopodia and F-actin bundles (*p* ≤ 0.05). mtROS suppression with H_2_O_2_ scavengers and mitochondrial-specific antioxidants significantly decreased migratory structures and reversed G1P3-induced migration and invasion (*p* ≤ 0.05). Knocking down G1P3 decreased both migration and migratory structures in MCF-7^G1P3^ cells. Moreover, gene networks involved in redox regulation, metastasis and actin remodelling were upregulated in MCF-7^G1P3^ cells.

**Conclusions:**

G1P3-induced mtROS have a direct role in migratory structure formation and nuclear gene expression to promote breast cancer cell metastasis. Therefore, interrupting mitochondrial functions of G1P3 may improve clinical outcomes in breast cancer patients.

## Introduction

G1P3 (ISG 6–16), one of the interferon (IFN)-stimulated genes (ISGs), was reported as antiapoptotic in myeloma, gastric and breast cancers.^1–4^ It belongs to the FAM14 protein family and is localised in mitochondria.^2, 3, 5, 6^ In G1P3-overexpressing cells, preservation of mitochondrial membrane potential (ΔΨ) was suggested to antagonise TRAIL-, IFNs- and chemotherapeutic-induced intrinsic apoptosis.^6^ Although G1P3 originally was identified as an ISG, our studies demonstrated its induction by 17β-estradiol in oestrogen receptor positive (ER+) breast cancer cells.^3^ Consequently, several lines of evidence illustrated a direct role of G1P3 in breast cancer development and progression. For example, ectopically expressed G1P3 lead to tamoxifen resistance in ER+ breast cancer cell line MCF-7, inhibited anoikis (detachment-induced apoptosis) to form hyperplasia in nontumourigenic mammary epithelial cell line MCF10A and silencing of G1P3 caused apoptosis of BT-549 cells as well as reduced the growth of MCF-7 cells.^3^

While improved diagnosis and therapies delayed ER+ breast cancer progression, the 5-year survival rates of metastatic breast cancers still remain ~22% and >90% of breast cancer-related deaths result from metastasis.^7–9^ Therefore, a better understanding of molecular mechanisms of metastasis are indispensable for curative outcomes. Recent studies have challenged the existing paradigms of metastasis and ascribed a prominent role for mitochondrial reactive oxygen species (mtROS) in metastasis.^10–12^ While a burst of ROS may induce apoptosis, low-to-moderate levels of ROS were suggested to augment cell survival, proliferation, migration and invasion.^13–15^ Subsequently, augmented levels of antioxidants were suggested to protect cells from the adverse effects of ROS. However, recent studies implicated that antioxidants counteract the anticancer effects of ROS to promote cancers.^14^ Taken together, a complex role for ROS emerges in cancer cells. Since G1P3 was localised in mitochondria and its elevated expression was associated with poor prognosis,^3^ its role in breast cancer metastasis was investigated with the hypothesis that G1P3-induced mitochondrial redox deregulation confers metastatic potentials in breast cancer cells.

## Materials and methods

### Cell lines

MCF-7^Vector^ and MCF-7^G1P3^ cells were maintained as described.^3^ The MDA-MB 231^Vector^ and MDA-MB 231^G1P3^ cells were engineered as described.^2, 3^ In brief, MDA-MB 231 cells (ATCC Manassas, VA, USA) were transduced with either PQCXIP (Clontech Inc, USA) or PQCXIP-G1P3 virions and stable transductants were selected in 1 μg/ml of puromycin.

### Antibodies

The rabbit anti-G1P3,^3^ mouse anti-Bcl2 (Zymed Inc), anti-AIF, anti-BAX and anti-Tubulin (Cell Signaling) antibodies were used at 1:1000 dilution. The rabbit anti-cytoskeleton actin (Fisher) was used at 1:5000 dilution. The goat anti-rabbit HRP conjugate (Bio-Rad) and the goat anti-mouse HRP conjugate were used as secondary antibodies with 1:10000 dilutions.

### Submitochondrial fractionation

Mitochondria were isolated from 8 × 10^6^ MCF-7^Vector^ and MCF-7^G1P3^ cells by using mitochondrial fractionation kit as described.^2, 3^ The mitochondrial matrix (MM), outer- and inner-mitochondrial membrane (OMM and IMM) were fractionated using differential centrifugation.^16^ In brief, isolated mitochondria were re-suspended in 12% digitonin for 15 min and centrifuged at 12,000 × *g* for 5 min at 4 °C. The supernatant contained OMM and the pellet containing IMM and MM was lysed in 2% Triton X-100 for 15 min and ultracentrifuged at 400,000 × *g* for 60 min at 4 °C. The triton-insoluble pellet was collected as IMM, and the triton-soluble supernatant was collected as the MM.

### Wound healing assay

To measure cell migration, 1.0 × 10^6^ cells were seeded in a 12-well plate and incubated for 24 h. Once the cells were confluent, a scratch was made using a pipette tip and cells were allowed to migrate for another 24 h. Effects of PEG-catalase on cell migration were determined by seeding 2.0 × 10^5^ cells in each well of a 24-well plate and treating with 250 U of PEG-catalase (Sigma-Aldrich Inc.) for 30 min before making the wound. Images of each wound were taken immediately after making the wound (0 h) and at indicated time points. The percent wound closure was determined by comparing the area of each wound at 0 h and at end point using NIH ImageJ programme.^17^

### Boyden chamber invasion assay

To determine the invasive potentials of MCF-7^Vector^ and MCF-7^G1P3^, 12-well transwell polycarbonate membrane inserts (8.0 μm pore size, Corning Inc., USA) were coated with 100 μl of 5% Matrigel (Invitrogen Inc., USA) and incubated overnight at 37 °C. Next day, 2.5 × 10^5^ cells were seeded on top of Matrigel in complete media. The transwell chambers were then placed in wells containing 750 μl of complete media and cells were allowed to invade through the Matrigel for 72 h. At the end of incubation, the Matrigel was removed, the membrane was washed with 1× PBS, the non-invaded cells were cleared from membrane using a cotton swab, and invaded cells were stained with 0.1% crystal violet and counted.

### Mitochondrial ROS measurement

For measuring mitochondrial ROS levels, 1.0 × 10^5^ cells were seeded on a coverslip in a 6-well plate and allowed to adhere for 24 h. Then, cells were loaded with 50 nM of either MitoTracker®Red (CM-XRos, Invitrogen Inc., USA) or reduced MitoTracker®Red (CM-H2Xros, Invitrogen Inc., USA) for 40 min, fixed with 100% ice-cold methanol for 15 min and imaged.

### ROS scavenging

For scavenging, ROS cells were treated with either antioxidant *N*-acetyl cysteine (NAC) (2.5 μM) or MitoTEMPO (2.5 μM) in plain media for the indicated time in actin remodelling, migration and invasion assays. Immunofluorescent-stained cells were mounted using Prolong gold antifade reagent with nuclear stain DAPI (Invitrogen Inc., USA).

### Actin cytoskeletal staining

For visualising F-actin, 5.0 × 10^5^ cells were seeded on coverslips in a 6-well plate for 24 h, fixed with 4% paraformaldehyde for 6 min and permeabilised using 0.1% Triton X-100 for 6 min. Cells were then incubated with 2.5 µM rhodamine phalloidin for 45 min and mounted using Prolong Gold antifade reagent with DAPI. Stained cells were imaged using Olympus BX51 fluorescence microscope with ×100 objective and fluorescence intensities were analysed using ImageJ software. Migratory structures including F-actin arcs (a curve-like formation of F-actin towards the leading edge of the motile cell), lamellipodia (a thin cytoplasmic sheet that extends to the front edge of the moving cells), filopodia (finger-like protrusions beyond lamellipodia) and bundles (thick supportive structures present within filopodia) of 50 images of either MCF-7^Vector^ and MCF-7^G1P3^ cells were enumerated manually.

### Knockdown of G1P3 expression

G1P3 knockdown with siRNA was done as described.^2, 3^

### Microarray analysis

Microarray analysis was performed at the University of Chicago Genomics Facility using Illumina HumanHT-12_V4_0 Expression BeadChip. Average signal and detection *p* values of Direct Hyb expression data was obtained using Illumina GenomeStudio Gene Expression (GX) Module (Illumina Inc.) and were imported into Arraystar expression analysis software version 15.0.1 (DNASTAR Inc.). Genes with average signal >10 were selected for determining differential expression and hierarchical clustering.

### Microscopy and imaging

The bright field images of invasion assays were captured using Zeiss AxioVert A1 inverted microscope (Zeiss Inc.) and Moticam Pro 282B CCD camera with Motic Image Plus vs 2.0 software (Motic Inc.) at ×10 magnification. The fluorescence images were captured using Olympus BX51 microscope with ×100 objective and Jenoptik ProgRes® Mf^Cool^ monochrome CCD camera (Jenoptik Inc.). Confocal imaging was performed using Olympus FV3000 microscope with ×60 objective lens with oil immersion. An optical zoom of ×2 optical zoom was applied and a PMT of 700 V and laser power of 52% for red channel (Alexa Flour 568) was maintained. The Z-stack images were acquired using 0.5 µm step size and each section was imaged three times for averaging. Mean fluorescence intensity and wound closure were calculated by using ImageJ or Fiji software.^17^

### Statistical analysis

One-way ANOVA and *t*-tests were performed with GraphPad Prism v6.0 (Graphpad software Inc.). Results were expressed as mean ± SEM and a *p* value <0.05 was considered significant.

## Results

### Distant metastasis-free survival (DMFS) is reduced in breast cancer patients with high G1P3 expression

**Fig. 1:**
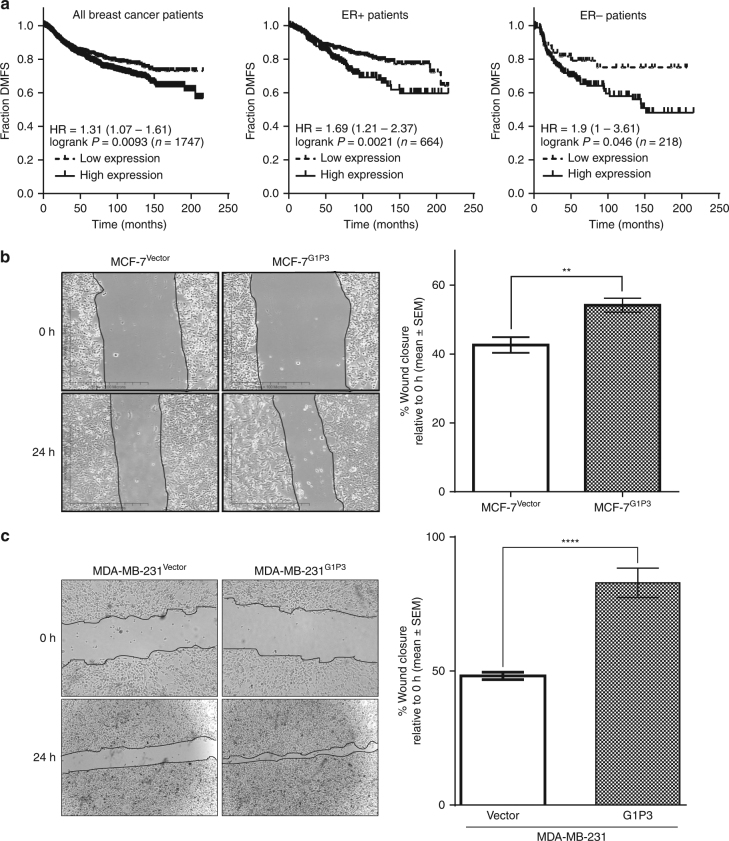
Elevated G1P3 expression is associated with poor DMFS and augmented migration of breast cancer cells. **a** Elevated G1P3 was significantly associated with poor DMFS prognosis in all (*n* = 1747), ER+ (*n* = 664) and ER− (*n* = 218) breast cancer patients in Kaplan–Meier survival analysis. A *p* value of 0.05 was considered significant. **b**, **c** Constitutively expressed G1P3 promoted breast cancer cell migration. In wound healing assays, G1P3-expressing cells migrated faster than vector-expressing MCF-7 (*p* = 0.0013) and MDA-MB 231 cells (*p* ≤ 0.0001). Images are representative of three independent experiments and each bar on the graph is mean ± SEM of three independent experiments, **p ≤ 0.01 and ***p ≤ 0.001 (*t*-test)

We previously reported the association between elevated G1P3 expression and poor relapse free (RFS) and overall survival (OS) in ER+ breast cancer patients.^3^ Since there was a limited number of DMFS cases, G1P3’s effect on DMFS was unclear. To overcome this limitation, in the current study, we employed KM plotter (http://www.kmplot.com), a publicly available database portal with 5143 breast cancer cases including 1747 DMFS cases.^18^ Analyses of the KM plot data sets identified a significant association between high G1P3 expression and poor DMFS in breast cancer with a hazard ratio (HR) of 1.31, *p* ≤ 0.05 (Fig. 1a, left panel). Moreover, G1P3 was associated with poor DMFS in both ER+ (*n* = 664) and in ER− (*n* = 218) with an HR of 1.69 (*p* = 0.0021) and 1.9 (*p* = 0.046), respectively (Fig. 1a). Upregulated G1P3 was also associated with poor prognosis in other clinical databases. A search in Prognoscan, a database with large collection of cancer microarray data sets with patient prognosis information^19^ also identified the association of high G1P3 expression with poor prognosis in 22 cancer studies (data not shown) and poor DMFS in two breast cancer studies (Supplemental Fig. 1A). The first data set, GSE11121, consisted of 78% ER+ cases (*n* = 200) and high G1P3 expression had an HR of 1.43 and the second data set contains 136 ER+ breast cancer patients who received tamoxifen as adjuvant therapy had an HR of 1.55 in high G1P3 cases (*P* = 0.001454). Taken together, these results implicate a role for G1P3 in promoting breast cancer metastasis.

### Stably expressed G1P3 promoted the migration of MCF-7 (ER+) and MDA-MB 231 (ER−) cells

Since cell migration is a prerequisite for metastasis, effects of elevated G1P3 expression on the migration of ER+ (MCF-7) and ER− (MDA-MB 231) cells were determined. In wound healing assays, at 24 h, vector-expressing MCF-7 (MCF-7^Vector^) cells closed 42.6 ± 2.3% of the wound whereas, stably expressed G1P3 cells (MCF-7^G1P3^), closed 54.2 ± 2.0% of the wound (Fig. 1b). These results suggested that MCF-7^G1P3^ migrated 1.3-fold faster than the MCF-7^Vector^ cells (*p* = 0.0013). Next, G1P3’s effect on ER− breast cancer cells were determined by comparing the migration rates of vector (MDA-MB 231^Vector^) and G1P3-expressing cells (MDA-MB 231^G1P3^). As we reported earlier, MDA-MB 231 cells lack basal G1P3 expression^3^ and transduction of G1P3 expression cassette resulted in the expression of 13 kD protein that was detected using G1P3-specific antibody (Supplemental Fig. 1B). Since MDA-MB 231 cells migrate faster, a wider wound was created to assess the wound closure. At 24 h, MDA-MB 231^Vector^ cells closed 48.2 ± 1.4% of the wound and during the same period MDA-MB 231^G1P3^ cells migrated significantly faster and resulted in 82.9 ± 5.5% of wound closure (*p* ≤ 0.0001, Fig. 1c). These results suggested that aberrant upregulation of G1P3 results in increased migration of both ER+ and ER− breast cancer cells. Since G1P3 is a target of oestrogen signalling,^3^ further analyses were carried out in ER+ breast cancer cell line MCF-7.

A moderate increase in ROS could promote cell growth and proliferation,^14, 15^ which could impact cell migration. Therefore, growth and migration of MCF-7^Vector^ and MCF-7^G1P3^ cells over a period of 96 h were determined. Compared to MCF-7^Vector^, MCF-7^G1P3^ cells migrated significantly faster and closed the wound 19.8%, 38.3%, 72.7% and 94.7%, respectively, at 24, 48, 72 and 96 h (Supplemental Figs. 1C and 1D, *p* ≤ 0.05). In growth assays, both MCF-7^Vector^ and MCF-7^G1P3^ cells exhibited similar growth rates at 24, 48, 72 and 96 h (Supplemental Fig. 1E). Taken together, these results suggest that the increased rates of migration and invasion of MCF-7^G1P3^ cells were not due to augmented proliferation.

### G1P3 increased the mitochondrial reactive oxygen species (mtROS) levels in MCF-7 cells

**Fig. 2:**
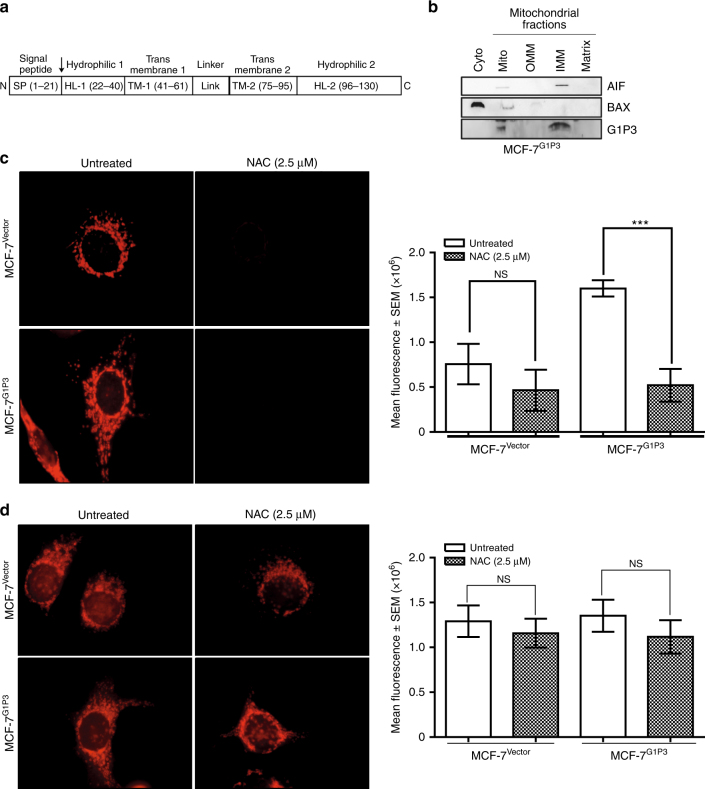
Mitochondrial reactive oxygen species (mtROS) were elevated in MCF-7^G1P3^ cells. **a** Schematics of structural features of G1P3 and amino acid positions. Domain analysis of G1P3 identified a 21 amino acids (a.a) putative mitochondrial signal peptide (SP) in its N terminus; followed by a 19 a.a. hydrophilic domain (HL1); a 20 a.a. transmembrane region (TM1); a linker region (L) that connects TM1 with a 20 a.a TM2 domain; and a 34 a.a hydrophilic-2 (HL2) region. **b** G1P3 is localised on the inner-mitochondrial membrane (IMM). Immunoblot analysis of cytoplasmic (cyto), mitochondrial (mito) outer mitochondrial membrane (OMM), IMM and mitochondrial matrix (matrix) fractions of MCF-7^G1P3^ cells with G1P3-specific antibody detected its localisation in mitochondrial and IMM fractions. Fractionation of AIF and BAX were used for assessing the purity of cytoplasmic, mitochondrial and IMM fractions. **c** G1P3 overexpression significantly increased mtROS. The mtROS in untreated and NAC-treated cells were measured using reduced MitoTracker red (CM-H2Xros) that fluoresces only in actively respiring mitochondria. Images at ×100 were acquired using Olympus BX51 microscope and mean fluorescence intensity of each cell was calculated using ImageJ software (*p* = 0.0004). **d** MCF-7^Vector^ and MCF-7^G1P3^ cells have similar number of mitochondria. Fluorescence from the total number of mitochondria in MCF-7^Vector^ and MCF-7^G1P3^ cells was assessed using oxidised MitoTracker red (CM-Xros). Each image is a representative of six independent experiments and each bar on the graph (left panel) is mean ± SEM of 120 cells, NS = *p* > 0.05, **p ≤ 0.01 (ANOVA)

Although G1P3 was identified to be a mitochondrial protein with IFI6 domain,^2–4^ its submitochondrial localisation was unknown. The primary structure of G1P3 consists of a signal peptide in its N terminus (Fig. 2a). Therefore, mitochondria from MCF-7^G1P3^ cells were subfractionated into outer- (OMM) and inner-mitochondrial membrane (IMM), and mitochondrial matrix (MM). Consistent with our previous report,^3^ in immunoblot analysis, most of G1P3 was localised in the mitochondrial fractions (Fig. 2b). Among submitochondrial fractions, IMM fractions had most of G1P3 with undetectable levels in OMM and MM fractions (Fig. 2b). Reprobing of immunoblot membrane with AIF (a marker of IMM) and BAX (a marker for cytoplasmic and OMM) identified their localisation in appropriate submitochondrial fractions, indicating the near purity of fractions employed (Fig. 2b).

The IMM harbours electron transport complexes, the main site of mtROS production.^20^ Since mtROS is associated with various cellular processes including cancer cell metastasis,^21, 22^ G1P3’s effects on mtROS levels were tested. Both MCF-7^Vector^ and MCF-7^G1P3^ cells were stained with reduced form of MitoTracker®Red (CM-H2Xros, Thermo Fisher Scientific) that fluoresces upon oxidation in an actively respiring mitochondrion. Compared to vector control cells, MCF-7^G1P3^ cells had a 2.1-fold higher CM-H2Xros mean fluorescence intensity (Fig. 2c). To confirm that increased CM-H2Xros fluorescence in MCF-7^G1P3^ was indeed due to mtROS, effects of ROS scavenger *N*-acetyl cysteine (NAC) on CM-H2Xros fluorescence was tested. While NAC reduced the CM-H2Xros fluorescence in MCF-7^Vector^ cells by 1.6-fold, it had a more pronounced effect in MCF-7^G1P3^ cells and resulted in a threefold reduction of mean fluorescence (*p* = 0.0004). Similarly, MitoTEMPO, a mitochondrial-targeted antioxidant that scavenge mitochondrial superoxide, also suppressed CM-H2Xros signals in MCF-7^Vector^ and MCF-7^G1P3^ cells (Supplemental Figs. 2A and 2B). Since an increase in the number of mitochondria could lead to an increased mean fluorescence intensity, total number of mitochondria in MCF-7^Vector^ and MCF-7^G1P3^ cells were compared using the oxidised form Mitotracker Red (CM-Xros) that emits fluorescence irrespective of the respiration status of mitochondria. In comparison analyses, both MCF-7^Vector^ and MCF-7^G1P3^ cells had similar levels of CM-Xros fluorescence (Fig. 2d), suggesting that increased CM-H2Xros-fluorescence in MCF-7^G1P3^ cells was the result of high mtROS levels but not a result of increased number of mitochondria.

### G1P3-induced mtROS promoted MCF-7 cell migration

**Fig. 3:**
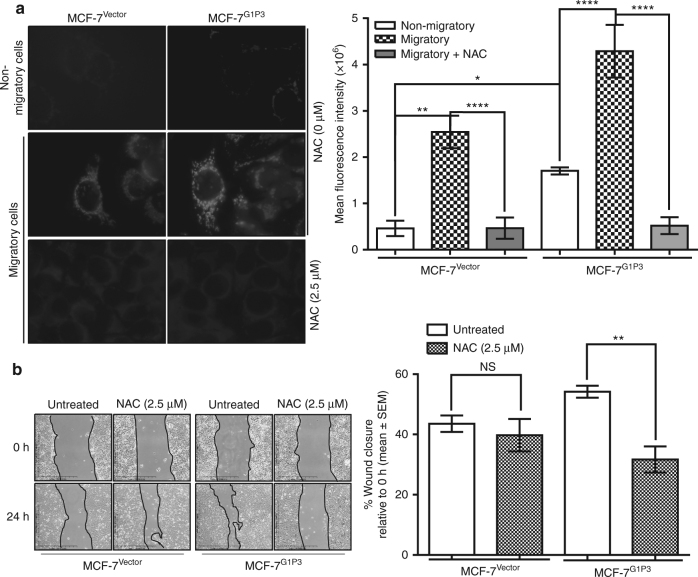

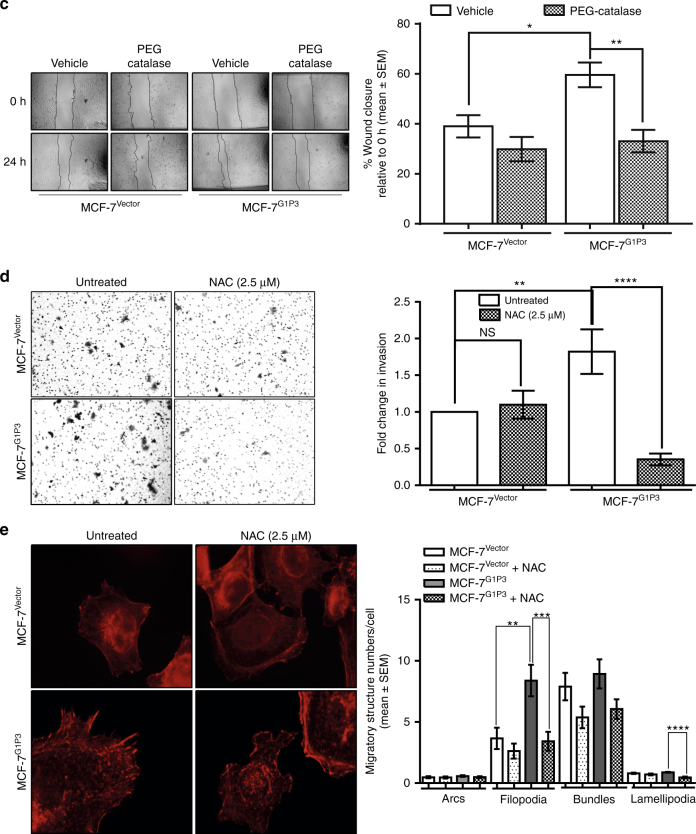
(Continued)

To test whether G1P3-induced mtROS mediate MCF-7^G1P3^ cell migration, mtROS levels in migratory vs nonmigratory cells as well as the effects of suppressing mtROS on cell migration was determined. The mtROS levels in actively migrating MCF-7^Vector^ and MCF-7^G1P3^ cells were determined by comparing mean CM-H2Xros fluorescence intensity in migrating (at the centre of wound) to nonmigrating cells (behind the wound border). Relative to nonmigrating cells, migrating MCF-7^G1P3^ cells had 4.4-fold more mtROS, whereas migrating MCF-7^Vector^ cells had 2.6-fold more mtROS than the nonmigrating MCF-7^G1P3^ cells (*p* ≤ 0.0001, Fig. 3a). The mitochondria of migrating MCF-7^G1P3^ had 1.7-fold more mtROS than that of migrating MCF-7^Vector^ cells (*p* ≤ 0.0001, Fig. 3a). Moreover, both NAC and MitoTEMPO suppressed mtROS in migrating MCF-7^Vector^ and MCF-7^G1P3^ cells (Fig. 3a and Supplemental Figs. 2A and 2B).

Because NAC pretreatment lowered the mtROS to basal levels in MCF-7^Vector^ and MCF-7^G1P3^ cells (Fig. 3a, bottom panels), effects of mtROS suppression with NAC on cell migration were tested. While NAC pretreatment had no significant effect on MCF-7^Vector^ cell migration, it significantly reduced the migration rate in MCF-7^G1P3^ cells (1.7-fold relative to untreated cells, *p* = 0.0065, Fig. 3b). In MCF-7^G1P3^ cells, NAC pretreatment reduced the wound closure from 54.2 ± 2.0 to 31.7 ± 4.3% at 24 h. This was lower than the closure of wound in NAC-pretreated MCF-7^Vector^ cells (Fig. 3b, right panel).

NAC was suggested to scavenge non-free radicals such as hydrogen peroxide (H_2_O_2_).^23^ Therefore, role of H_2_O_2_ in mediating G1P3-induced migration of MCF-7 cells was tested using polyethylene glycol-modified catalase (PEG-catalase), which catalyses the decomposition of H_2_O_2_.^24^ While PEG-catalase affected the migration of MCF-7^Vector^ cells marginally (39 ± 4.5% vehicle vs 29.9 ± 4.9% PEG-catalase), it significantly reduced (1.8-fold) wound closure in MCF-7^G1P3^ cells (Fig. 3c, *p* = 0.0017). PEG-catalase reduced the wound closure from 59.6 ± 5.0 to 33.1 ± 4.5% at 24 h (Fig. 3c)

### G1P3 augmented the invasive potentials of MCF-7 cells

Besides migration, the ability to invade through extracellular matrix is indispensable for cancer cell metastasis. Therefore, G1P3’s effects on the invasive potentials of MCF-7 cell were assessed using Boyden chambers coated with Matrigel as basement membrane matrix material. Relative to vector control cells, 1.8-fold more number of MCF-7^G1P3^ cells invaded through the Matrigel at 72 h (*p* ≤ 0.001, Fig. 3d). Since MCF-7^G1P3^ cell migration was mtROS dependent, the effect of mtROS in mediating MCF-7^Vector^ and MCF-7^G1P3^ cell invasion was assessed. For this, cells were treated with NAC 4 h after adhering to Matrigel, then invasion through Matrigel was allowed to proceed for 72 h. Both untreated and NAC-treated MCF-7^Vector^ cells invaded through the Matrigel at similar rates (Fig. 3d). However, NAC pretreatment significantly reduced the invasion rate of MCF-7^G1P3^ cells (*p* ≤ 0.0001). Compared to cells left untreated, NAC reduced the invasion of MCF-7^G1P3^ cells by fivefold (Fig. 3d). Moreover, NAC markedly lowered the invasion rate of MCF-7^G1P3^ cells than MCF-7^Vector^ cells.

### G1P3-induced mtROS remodelled cytoskeletal actin and increased migratory structures in MCF-7 cells

Cytoskeletal remodelling is indispensable for cell migration and invasion and recent studies have implied a role for mtROS in actin remodelling.^21, 22, 25^ To test the effect of G1P3-induced mtROS on cytoskeletal actin, F-actin levels in intact cells were determined using phalloidin staining. In MCF-7^G1P3^ cells, the F-actin was more concentrated near plasma membrane, whereas it was found to be dispersed in MCF-7^Vector^ cells (Fig. 3e, left panels). Additionally, NAC treatment markedly reduced phalloidin signals and altered actin organisation in both MCF-7^Vector^ and MCF-7^G1P3^ cells, suggesting a role for mtROS in actin dynamics and organisation in these cells (Fig. 3e, right panels).

Actin remodelling was suggested to drive migratory structure formation at the leading edges of a migrating cell.^26–28^ Therefore, effects of G1P3-induced mtROS on migratory structures were determined by comparing the number of F-actin arcs, filopodia, bundles and lamellipodia in MCF-7^Vector^ and MCF-7^G1P3^ cells. Both MCF-7^Vector^ and MCF-7^G1P3^ had similar number of arcs, bundles and lamellipodia (Fig. 3e). However, MCF-7^G1P3^ cells had a significantly higher number of filopodia than the MCF-7^Vector^ cells (*p* = 0.0008, Fig. 3e). On average, MCF-7^Vector^ cells had 3.7 ± 0.9 filopodia per cell, whereas MCF-7^G1P3^ cells had 8.4 ± 1.3 filopodia per cell. Since MCF-7^G1P3^ cell migration was dependent on ROS, its effects on migratory structures were assessed after suppressing ROS with NAC. While NAC had a minimal effect on migratory structures in MCF-7^Vector^, it reduced the number of filopodia and lamellipodia by 2.5- and 2.0-folds, respectively, in MCF-7^G1P3^ cells (*p* < 0.0001, Fig. 3e).

### G1P3-induced mtROS controlled filopodia and actin bundles in MCF-7 cells undergoing active migration

**Fig. 4:**
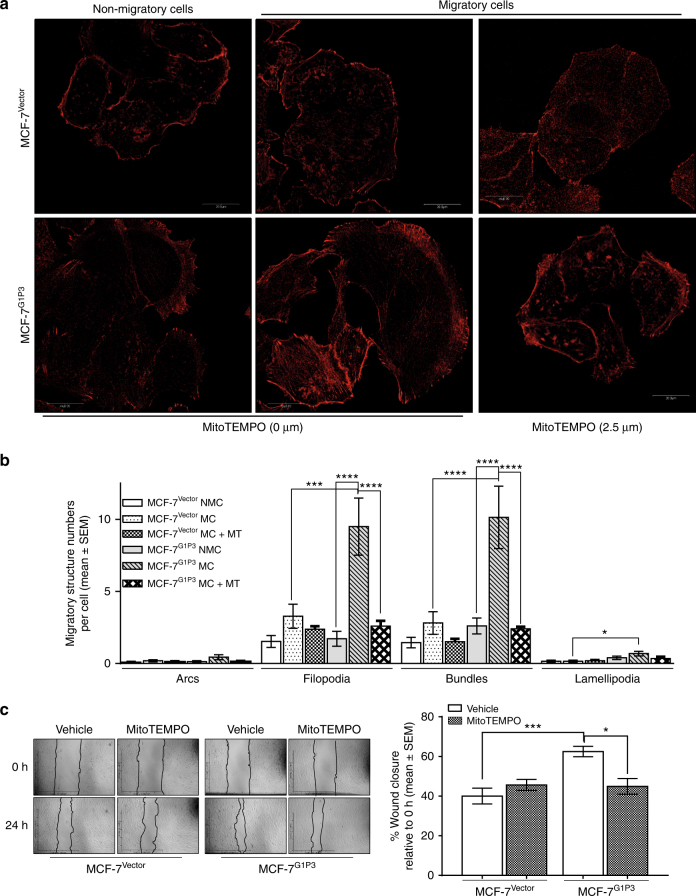
The mtROS scavenger MitoTEMPO significantly reduced the number of migratory structures in migrating MCF-7^G1P3^ cells. **a** Abrogation of G1P3-induced mtROS with MitoTEMPO significantly reduced migratory structures in migrating MCF-7^G1P3^ cells. Migratory structures of untreated and MitoTEMPO-treated MCF-7^Vector^ and MCF-7^G1P3^ cells were detected with actin staining. Images of migrating and nonmigrating cells were captured with Olympus BX61 confocal microscope at ×60 magnification and analysed using ImageJ software. Each image is a representative of three independent experiments. **b** Enumeration of migratory structures. Migratory structures in 25 cells were enumerated and represented as the average number of migratory structures per cell (*p* ≤ 0.0001). Each bar on the graph is mean ± SEM and a *p* value of 0.05 in ANOVA was considered as significant. **c** MitoTEMPO significantly reduced augmented migration of MCF-7^G1P3^ cells. Migration rate of MCF-7^Vector^ and MCF-7^G1P3^ cells left untreated or pretreated with MitoTEMPO was determined in wound healing assays. Each image is a representative of three independent experiments and each bar on the graph is mean ± SEM of three independent experiments. NS = *p* > 0.05, **p ≤ 0.01, ***p ≤ 0.001, ****p ≤ 0.0001 (ANOVA)

To determine whether G1P3-induced mtROS control migratory structures in cells undergoing active migration, actin-dependent migratory structures in nonmigratory and migratory cells were determined. A marginal increase in the number of filopodia (2.14-fold) and F-actin bundles (1.94-fold) were observed in MCF-7^Vector^ cells undergoing migration (Fig. 4a, b). However, compared to nonmigrating cells, both filopodia (5.5-fold) and actin bundles (3.9-fold) were increased in migrating MCF-7^G1P3^ cells (*p* < 0.001, Fig. 4a, b). When MCF-7^Vector^ and MCF-7^G1P3^ cells were treated with MitoTEMPO, actin arcs and lamellipodia remained similar in number as that of untreated control cells, but, there was a significant reduction in the number of filopodia and actin bundles in migrating MCF-7^G1P3^ cells (*p* ≤ 0.0001, Fig. 4b).

Since MitoTEMPO reduced the number of migratory structures in MCF-7^G1P3^ cells, its effects on cell migration were tested. Four-hour pretreatment with MitoTEMPO reduced the migration rate of MCF-7^G1P3^ cells but not MCF-7^Vector^ cells (Fig. 4c). Untreated MCF-7^G1P3^ cells closed 62.5 ± 2.6% of wound at 24 h and MitoTEMPO decreased MCF-7^G1P3^ migration to the basal level of vector control cells and resulted in a wound closure of 44.9 ± 3.9% (*p* = 0.0113).

### Downregulating G1P3 abrogated augmented migration and migratory structures in MCF-7^G1P3^ cells

**Fig. 5:**
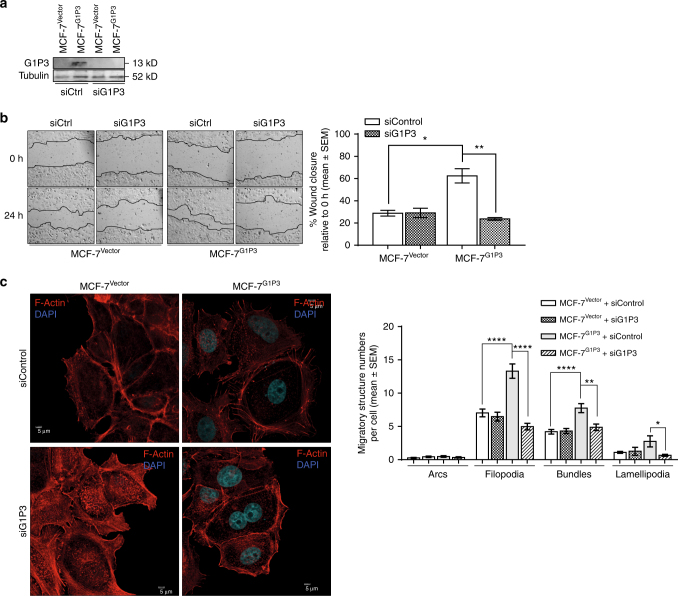
Abrogation of G1P3 expression reversed migratory phenotype of MCF-7^G1P3^ cells. **a** Downregulation of G1P3 expression in MCF-7^G1P3^ cells. MCF-7^Vector^ and MCF-7^G1P3^ cells were transfected with either scrambled (siCtrl) or G1P3 (siG1P3) siRNAs and G1P3 protein expression was assessed after 48 h. Immunoblot image is a representative to two independent experiments. **b** Abrogation of G1P3 expression reversed enhanced migration of MCF-7^G1P3^ cells. Forty-eight after transfection of siRNAs, migration rate of MCF-7^Vector^ and MCF-7^G1P3^ cells was assessed. Each image is representative of three independent experiments done in duplicates and each bar on the graph (right panel) is mean ± SEM. **c** G1P3 downregulation abrogated increased number of migratory structures in MCF-7^G1P3^ cells. Migratory structures of siCtrl and siG1P3 transfected MCF-7^Vector^ and MCF-7^G1P3^ cells were detected by actin staining and confocal microscopy. Images of migrating cells were captured with Olympus FV3000 confocal microscope at ×100 magnification and analysed using ImageJ software. Each image is a representative of three independent experiments and each bar on the graph is mean ± SEM of 25 cells

To test whether augmented migration of MCF-7^G1P3^ cells was the direct result of elevated expression of G1P3, its expression in MCF-7^G1P3^ cells was downregulated with G1P3-specific siRNA. While scrambled siRNA (siCtrl) had no effect on G1P3 protein levels, G1P3-specific siRNA (siG1P3) abrogated G1P3 expression in MCF-7^G1P3^ cells (Fig. 5a). In wound closure assays, migration of MCF-7^G1P3^ cells was unaffected by scrambled siRNAs (Fig. 5b). While migration of MCF-7^Vector^ cells was unaffected by siG1P3, it significantly reduced the migration of MCF-7^G1P3^ cells (*p* = 0.0088). At 24 h, MCF-7^G1P3^ cells transfected with scrambled siRNA closed the wound by 62.5 ± 6.4%, whereas siG1P3 reduced the migration of MCF-7^G1P3^ cells by 2.6-fold to result in 23.7 ± 1.3% wound closure.

Next, effects of G1P3 knockdown on migratory structures in MCF-7^G1P3^ cells were assessed. As identified in Figs. 4 and 5, compared to MCF-7^Vector^, both filopodia and actin bundles were significantly higher in MCF-7^G1P3^ cells with control siRNA (*p* < 0.0001), suggesting that migratory structures are unaffected by scrambled siRNA. However, abrogating G1P3 expression with siG1P3 significantly reduced the number of filopodia, actin bundles and lamellipodia (Fig. 5c). In MCF-7^G1P3^ cells, siG1P3 reduced the average number of filopodia (13.3 ± 1.1–4.9 ± 3.1), actin bundles (7.8 ± 0.7–4.9 ± 0.5) and lamellipodia (2.8 ± 0.8–0.7 ± 0.2). Taken together, these results suggest that enhanced migration and increased number of migratory structures in MCF-7^G1P3^ cells was due to elevated G1P3 expression.

### Metastasis- and redox-associated genes are upregulated in MCF-7^G1P3^ cells

**Fig. 6:**
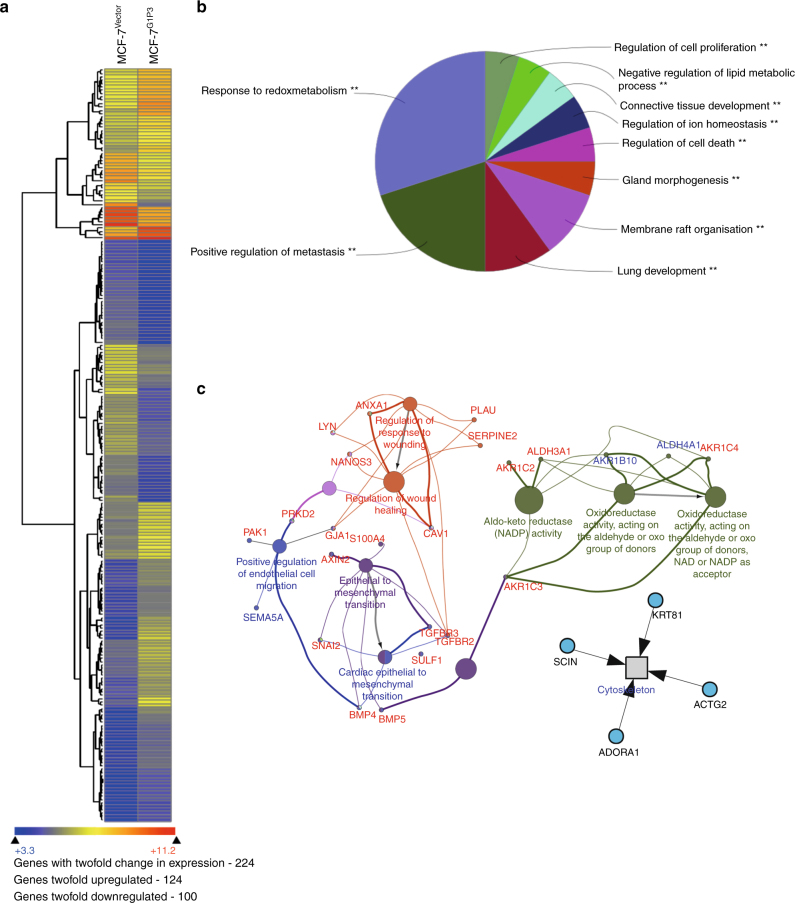
Genes involved in oxidative stress response and prometastatic networks are upregulated in MCF-7^G1P3^ cells. **a** Heat map of 224 differentially expressed genes in MCF-7^G1P3^ cells relative to MCF-7^Vector^ cells. **b** Over-represented ontological categories in MCF-7^G1P3^ cells. **c** Functionally organised GO/pathway term network was generated using ClueGo for genes upregulated ≥twofold in MCF-7^G1P3^ cells. GO terms with ≥0.4 kappa score are represented as nodes. Node size indicates term enrichment significance and partial overlap suggests functionally related groups or biological functions. Pathways are visualised using Cytoscape 3.4

The mtROS manifests its pleiotropic effects through a variety of cellular mechanisms including altered nuclear gene expression. To test whether G1P3-induced mtROS alter expression of metastasis-associated nuclear genes, gene expression profiles of MCF-7^Vector^ and MCF-7^G1P3^ cells were compared using Illumina HumanHT-12 v4 BeadChip array. Relative to MCF-7^Vector^ cells, 224 genes were differentially expressed (twofold or more) in MCF-7^G1P3^ cells (Fig. 6a). ClueGo analysis that integrates Gene Ontology (GO) terms^29, 30^ identified upregulation of gene networks involved in redox regulation, cell migration and epithelial-to-mesenchymal transition (EMT) and downregulation of actin remodelling networks in MCF-7^G1P3^ cells (Fig. 6b, c). Expression array results were further validated using quantitative RT-PCR (qRT-PCR) (Table 1). Among 22 upregulated genes investigated, four (AKR1B10, AKR1C2, AKR1C3 and AKR1C4) are regulators of oxidoreductase; seven are associated with EMT process (AXIN2, BMP4, BMP5, S100A4, SNAI2, TGFBR2 and TGFBR3) and 11 genes are associated with cell migration (PAK1, PRKD2, SEMA5A, ANXA1, LYN, NANOS3, PLAU, SERPINE2, GJA1, BMP4, and CAV1). Additionally, four genes (ABLIM1, LCP1, NEBL and SCIN) that bind to actin were downregulated in MCF-7^G1P3^ cells. These results further validate G1P3’s role in modulating mtROS and actin remodelling to promote breast cancer cell migration and invasion.

**Table 1:** Expression changes of genes associated with redox regulation and metastatic potential in MCF-7^G1P3^ cells

Gene symbol	Description	Fold change in MCF-7^G1P3^ cells		GO function
		Gene array	qRT-PCR	
AKR1C2	Aldo-keto reductase family 1 member C2	5.27	3.50 ± 0.77*	Aldo-keto reductase (NADP)
AKR1B10	Aldo-keto reductase family 1 member B10	4.45	5.15 ± 1.21*	Aldo-keto reductase (NADP) Oxidoreductase activity
AKR1C3	Aldo-keto reductase family 1 member C3	2.81	2.37 ± 0.43*	
AKR1C4	Aldo-keto reductase family 1 member C4	3.59	3.13 ± 0.46**	
AXIN2	Axin2	2.09	ND	Epithelial-to-mesenchymal transition
BMP4	Bone morphogenetic protein 4	2.03	ND	
BMP5	Bone morphogenetic protein 5	5.59	3.41 ± 0.72*	
S100A4	S100 calcium-binding protein A4	2.83	ND	
SNAI2	Snail family transcriptional repressor 2	2.18	2.06 ± 0.23**	
TGFBR2	Transforming growth factor beta receptor 2	4.90	2.96 ± 0.25**	
TGFBR3	Transforming growth factor beta receptor 3	4.61	ND	
PAK1	p21 (RAC1)-activated kinase 1	2.27	2.03 ± 0.02****	Positive regulation of endothelial cell migration
PRKD2	Protein kinase D2	2.53	ND	
SEMA5A	Semaphorin 5A	2.0	ND	
ANXA1	Annexin A1	6.76	5.46 ± 1.21*	Regulation of response to wounding Regulation of wound healing
LYN	LYN proto-oncogene, Src family tyrosine kinase	2.51	2.44 ± 0.28*	
NANOS3	Nanos C2HC-type zinc finger 3	2.40	ND	
PLAU	Plasminogen activator, urokinase	2.30	ND	
SERPINE2	Serpin family E member 2	2.75	2.08 ± 0.05****	
GJA1	Gap junction protein alpha 1	2.10	3.53 ± 0.72*	Regulation of response to wounding Regulation of wound healing
CAV1	Caveolin 1	4.52	4.75 ± 0.90*	
NEBL	Nebulette	0.48	ND	Actin binding and remodelling
LCP1	Lymphocyte cytosolic protein 1	0.45	ND	
ABLIM1	Actin-binding LIM protein 1	0.25	ND	
SCIN	Scinderin	0.06	0.248 ± 0.009***	

Real-time qRT-PCR results are mean ± SEM of three independent experiments, a *p* value of ≤0.05 are considered significant (* = ≤0.05; ** = ≤0.01; *** = ≤0.001; **** = ≤0.00001)

*ND* not determined

## Discussion

Identification of G1P3 as a poor prognostic factor in breast cancer challenged the dogma that upregulated ISGs in cancer cells are beneficial.^3^ Conforming to its localisation in mitochondria, G1P3 antagonised intrinsic apoptosis in a variety of cancer cells.^2, 4^ Antiapoptotic function of G1P3 could in part explain the association between G1P3 upregulation and poor RFS,^3^ but not poor DMFS identified in the current study (Fig. 1a and supplemental Figures. 1A and 1B). Conforming to our hypothesis, we demonstrate a direct role for G1P3-induced mtROS in promoting breast cancer cell migration and invasion by augmenting migratory structures and altering nuclear gene expression. This highlights an integral role for G1P3 in eliciting multiple mechanisms to augment breast cancer progression. Poor DMFS of breast cancer patients with high G1P3 expression further support this conclusion (Fig. 1a). In agreement with this, ectopically expressed G1P3 promoted the migration and invasion of both ER+ (MCF-7) and ER− (MDA-MB 231) breast cancer cells (Fig. 1b, c). Although prior studies identified G1P3 as a mitochondrial protein,^2–4^ this study defined its localisation on IMM of MCF-7^G1P3^ cells (Fig. 2b). Most (>90%) of IMM proteins, including G1P3, are encoded by nuclear genes.^31^ Although the precise mechanism of G1P3 import into mitochondria is unclear, the putative mitochondrial targeting signal peptide in its N terminus and two transmembrane motifs support its localisation on IMM (Fig. 2a). Consistent with its IMM localisation, mtROS was higher in G1P3-expressing cells. A burst of mtROS was implicated in opening of mitochondrial permeability transition pore (MPTP) to induce apoptosis and low-to-moderate mtROS levels were suggested to foster cancer progression.^14, 15^ Despite increased mtROS, mitochondria of G1P3-overexpressing cells were intact (Fig. 2c), slightly hyperpolarised and maintained the mtΔΨ under apoptotic stress.^2, 3^ These results suggested that in MCF-7^G1P3^ cells, mtROS levels were not high enough to open MPTP or mtROS-mediated apoptosis was neutralised. Considering G1P3 is induced by both IFN and oestrogen and both of these pathways elicit ROS production, it is likely that G1P3 may play a role in mediating immuno-endocrine-elicited redox signalling.

Accumulating evidence highlighted the importance of mitochondria in cancer cell metastasis.^11, 12, 22, 32^ Whether it was a mutation in NADH dehydrogenase subunit 6 (ND6),^32^ an overexpression of the survival protein survivin,^12^ or ETC overload and partial ETC inhibition,^11^ increased mtROS and associated redox deregulation was suggested as underlying mechanisms of metastasis promotion. This agrees with our results that increased G1P3-induced mtROS augments migratory and invasive potential of breast cancer cells that were attenuated by removal of mtROS with mitochondrial-specific antioxidants (Fig. 3). Moreover, PEG-catalase that decompose H_2_O_2_, reversed the augmented migration of MCF-7^G1P3^ cells, suggesting a role for H_2_O_2_ in mediating G1P3-induced migration (Fig. 3c). Compared to other ROS, H_2_O_2_ is non-radical, stable, diffusible and was suggested to mediate cell proliferation, survival and migration.^33–35^ Interestingly, NAC had more pronounced effects in suppressing MCF-7^G1P3^ cell migration and invasion than PEG-catalase and MitoTEMPO (Figs. 3 and 4). Since NAC is an efficient scavenger of H_2_O_2_ as well as hydroxy radicals (^•^OH), our current results suggest an involvement of other free radicals in the enhanced migration of MCF-7^G1P3^ cells.

Continuous and rapid remodelling of the actin microfilament is pivotal for cellular processes such as cell migration and invasion.^36^ A myriad of factors, including cytoplasmic ROS, were implicated in actin remodelling.^37^ Under pathophysiological states, actin was suggested as a direct target of oxidative modification by cytoplasmic ROS.^38–42^ However, the role of mtROS in this process remains unclear. Our results suggest that G1P3-induced mtROS remodels actin to increase migratory structures such as filopodia that were reversed by mtROS removal with antioxidants or by downregulating G1P3 protein (Figs. 3–5). Loss of migratory structures and reversal of augmented migration of MCF-7^G1P3^ cells by siG1P3 confirm the role of G1P3 in breast cancer cell migration (Fig. 5). F-actin turnover is also controlled by Ca_2_+-dependent actin severing and capping protein scinderin (SCIN).^43^ Decreased expression of SCIN in MCF-7^G1P3^ cells further support higher number of F-actin-containing migratory structures in MCF-7^G1P3^ cells (Fig. 6). Taken together, these results suggest a pleiotropic effect for G1P3-induced mtROS in actin remodelling to augment breast cancer cell migration.

Elevated mtROS usually leads to the opening of MPTP and depolarisation of mitochondria to maintain cells’ health.^44^ Contrary to this, even with increased mtROS, G1P3 cells had hyperpolarised mitochondria (increased mtΔΨ) and better survival under stress. Since mtROS were known to elicit changes in nuclear gene expression,^45, 46^ we postulated that G1P3-induced antioxidant genes blunt the toxic effects of high mtROS. In agreement with this postulation, network analysis and qRT-PCR identified enrichment of aldo-keto reductase (AKR1B10, AKR1C2, AKR1C3 and AKR1C4) and aldehyde dehydrogenase (ALDH3A1)-dependent antioxidant pathways in G1P3-expressing cells (Fig. 6b). Both aldo-keto reductase and aldehyde dehydrogenases were suggested to protect cells from oxidative damages through a variety of mechanisms.^47, 48^ Additionally, seven EMT-associated genes (AXIN2, BMP4, BMP5, S100A4, SNAI2, TGFBR2 and TGFBR3) and 11 migration regulators (PAK1, PRKD2, SEMA5A, ANXA1, LYN, NANOS3, PLAU, SERPINE2, GJA1, BMP4, and CAV1) were upregulated in MCF-7^G1P3^ cells (Fig. 6b and Table 1). While upregulation of antioxidant genes could be an adaptive response to G1P3-induced mtROS, induction of metastasis-associated genes further validates G1P3’s role in modulating mtROS-mediated redox signalling to promote breast cancer metastasis.

In summary, G1P3-induced mtROS manifests its pleiotropic effects through actin remodelling and by altering the expression of genes involved in EMT and cell migration. While exact mechanism of G1P3-induced metastasis is unclear, our results demonstrate that coordinated action of multiple pathways elicited by G1P3-induced mtROS augment F-actin-containing migratory structures to promote breast cancer cell migration and invasion. This may lead to poor DMFS in patients with high G1P3 expression and identify G1P3 as a negative prognosticator in breast cancer by promoting metastasis.

## Electronic supplementary material

Supplemental Figure 1A & 1B

Supplemental Figure 1C

Supplemental Figure 2A-2B

Supplemental Figure

## Availability of data and material

The expression array data that support the findings of current study are available at https://www.ncbi.nlm.nih.gov/geo/query/acc.cgi?acc=GSE113792.
