# Supplementary material for: G1P3 (IFI6), a mitochondrial localised antiapoptotic protein, promotes metastatic potential of breast cancer cells through mtROS
Source: Br J Cancer. 2018 Jun 14;119(1):52–64. doi: 10.1038/s41416-018-0137-3 (PMC6035266; doi:10.1038/s41416-018-0137-3)
Supplement: Supplementary file 4 — Supplemental Figure [file 41416_2018_137_MOESM4_ESM.docx]

**Supplemental Figure 1A.** **High G1P3 expression was associated with poor DMFS in Prognoscan database.** Two clinical studies (GSE11121 and GSE12093) in Prognoscan database with ER+ breast cancers showed significant association between high expression of G1P3 and DMFS. Patient characteristics, number of patients in each cohort and Kaplan-Mier plots are generated at [http: prognoscan.org](http://prognoscan.org/) . A p value of 0.05 was considered significant.

**Supplemental Figure 1B.** **Stable expression of G1P3 in MDA-MB-231 cells.** Whole cell lysates (30 μg) from MDA-MB 231^Vector^ and MDA-MB 231^G1P3^ cells were separated on a SDS gel and the expression of G1P3 was detected by immunoblot using anti- G1P3 antibody as described**.**^3^ β-actin was used as an internal control Image is a representative of 3 independent experiment which shown similar results.

**Supplemental Figure 1C. Augmented migration of migration of MCF-7^G1P3^ cells in time course studies.** Migration rate of MCF-7^Vector^ and MCF-7^G1P3^ cells were compared at 24, 48, 72, 96 hrs in migration assays after making a wider wound. Each image is a representative of 3 independent experiment done in duplicates and point on the line graph (bottom graphs) is Mean ± SEM, and level of significance was determined using ANOVA.

**Supplemental Figure 1D. MCF-7^Vector^ and MCF-7^G1P3^ cells have similar growth rate.** The growth of MCF-7^Vector^ and MCF-7^G1P3^ cells at 24, 48, 72, and 96 hrs were assessed using crystal violet assay. Each point on the line graph is Mean ± SEM of 3 independent experiments done in hexaplicates.

**Supplemental Figure 2A & B.** Mitochondrial reactive oxygen species (mtROS) were elevated in migrating MCF-7^G1P3^ cells. While cells were undergoing active migration in wound healing assay, the mtROS in non-migratory cells (behind the wound, top panels) and migrating (in the middle of wound, middle panel) were determined using reduced MitoTracker red (CM-H2Xros) and compared that to the MitoTEMPO (bottom panel) treated MCF-7^Vector^ and MCF-7^G1P3^ cells. Images at 100x were acquired using Olympus BX51 microscope and Mean fluorescence intensity of each cell was calculated using ImageJ software. Each image is a representative of 3 independent experiments and each bar on the graph (2B) is Mean ± SEM of 60 cells, NS = P > 0.05, **P ≤ 0.01, ***P ≤ 0.001, and ****P ≤ 0.0001 (ANOVA).
